# Grape Cane Extracts as Multifunctional Rejuvenating Cosmetic Ingredient: Evaluation of Sirtuin Activity, Tyrosinase Inhibition and Bioavailability Potential

**DOI:** 10.3390/molecules25092203

**Published:** 2020-05-08

**Authors:** Magdalena Anna Malinowska, Kévin Billet, Samantha Drouet, Thibaut Munsch, Marianne Unlubayir, Duangjai Tungmunnithum, Nathalie Giglioli-Guivarc’h, Christophe Hano, Arnaud Lanoue

**Affiliations:** 1 EA 2106 Biomolécules et Biotechnologies Végétales, UFR des Sciences Pharmaceutiques, Université de Tours, 31 av. Monge, F37200 Tours, France; magdalena.malinowska@univ-tours.fr (M.A.M.); kevin.billet@univ-tours.fr (K.B.); thibaut.munsch@univ-tours.fr (T.M.); marianne.unlubayir@univ-tours.fr (M.U.); nathalie.guivarch@univ-tours.fr (N.G.-G.); 2 Faculty of Chemical Engineering and Technology, Cracow University of Technology, 24 Warszawska St., 31-155 Cracow, Poland; 3 Laboratoire de Biologie des Ligneux et des Grandes Cultures, INRA USC1328, Université de Orléans, Pôle Universitaire d’Eure et Loire, 21 rue de Loigny-la-Bataille, F28000 Chartres, France; drouet.samantha@yahoo.fr (S.D.); duangjai.tun@mahidol.ac.th (D.T.); hano@univ-orleans.fr (C.H.); 4 Department of Pharmaceutical Botany, Faculty of Pharmacy, Mahidol University, Bangkok 10400, Thailand

**Keywords:** grape cane extracts, natural ingredients, polyphenols, *E*-resveratrol, *E*-ε-viniferin, sirtuin activation, tyrosinase inhibition, drug-likeness

## Abstract

Grape canes are waste biomass of viticulture containing bioactive polyphenols valuable in cosmetics. Whereas several studies reported the cosmetic activities of *E*-resveratrol, only few described the potential of *E*-ε-viniferin, the second major constituent of grape cane extracts (GCE), and none of them investigated GCE as a natural blend of polyphenols for cosmetic applications. In this study, we considered the potential of GCE from polyphenol-rich grape varieties as multifunctional cosmetic ingredients. HPLC analysis was performed to quantify major polyphenols in GCE i.e., catechin, epicatechin, *E*-resveratrol, *E*-piceatannol, ampelopsin A, *E*-ε-viniferin, hopeaphenol, isohopeaphenol, *E*-miyabenol C and *E*-vitisin B from selected cultivars. Skin whitening potential through tyrosinase inhibition assay and the activation capacity of cell longevity protein (SIRT1) of GCE were compared to pure *E*-resveratrol and *E*-ε-viniferin. Drug-likeness of GCE polyphenols were calculated, allowing the prediction of skin permeability and bioavailability. Finally, the present data enabled the consideration of GCE from polyphenol-rich varieties as multifunctional cosmetic ingredients in accordance with green chemistry practices.

## 1. Introduction

Conscious design of new skincare formulations based on natural ingredients has become a key issue in the cosmetics industry, in compliance with environmental responsibility. Botanical extracts are being selected according to their composition, biological activity, stability and skin permeability. These factors determine the overall efficiency of the ingredient and its cosmetic potential. Nowadays, there is a growing demand for new natural resources with effective skincare components that protect from stress sources including environmental pollution, harmful radiation as well as improper diet and stressful lifestyle [1]. Additionally, customers are looking for eco-friendly natural cosmetics, and plant residue-recycled biomolecules offer new perspectives towards sustainable sourcing.

Viticulture (and the wine sector) is one of the oldest and most developed industries in the world, where approximately 80% of grape fruits are used for winemaking [2]. It is well known that grape berries are a rich source of valuable compounds with health benefits such as anthocyanins, phenolic acids, flavan-3-ols, flavonols, proanthocyanidins, stilbenoids, melanin, fatty acids, minerals as well as vitamins [2]. Winemaking generates different biomolecule-rich byproducts, including pomaces (skin and seeds), lees, as well as other solid wastes like grape canes [3]. Among them, grape wood biomass, which are discarded after winter pruning, represent great potential for the development of new natural cosmetic ingredients due to a large abundance and the presence of polyphenols including stilbenoids [2,4]. Previous studies reported that grape varietal choice is determinant when developing polyphenol-rich GCE [4,5,6].

*E*-resveratrol, a well-known grape defense compound, exhibits several biological activities including antioxidant [7], anticancer [8,9], antifungal [10] and anti-inflammatory properties [11]. It also displays significant activity against the skin aging process through tyrosinase inhibition [12,13], which is a key mechanism to inhibit skin discoloration. The process of skin pigmentation is related to the presence of melanin and lipofuscin, of which excess and abnormal distribution in the skin cause dark spots. Uneven skin tone is one of the major symptoms of this aging process. Melanin is formed under the influence of tyrosinase during melanogenesis, regulates the biosynthesis of vitamin D3 and increases the skin’s resistance to sunburn and tumors [13,14]. When local hyperpigmentation occurs, besides aesthetic problems, it can also increase the risk of melanoma. Therefore, tyrosinase inhibitors like *E*-resveratrol can be attractive in the cosmetics and medicinal industries as depigmentation agents [15].

Additionally, the slowing down of skin aging processes can also occur through the activation of natural cell repairing mechanisms. It can be achieved by applying sirtuin-activating compounds [1]. A stimulation of SIRT1 activity, which maintains cell longevity, transcription factors and other DNA repairing proteins [16], has been reported to be crucial in the control of oxidative stress and in the regulation of aging process [17]. Sirtuins are normally regulated at transcription level, translation, protein stability and oxidation by natural inhibitors such as nicotinamide. Mammalian sirtuins like SIRT1, act as transcription regulators for the selected receptors and DNA repairing proteins. They also control energy metabolism, cell survival, DNA repair, tissue regeneration, inflammation mechanisms as well as neuronal signaling [1].

The presence of *E*-resveratrol and its derivatives in grape canes makes a great opportunity for the use of this natural cosmetic ingredient in the cosmetics industry as efficient anti-aging agents via sirtuin-activating and tyrosinase-inhibiting activities [1,16].

The requirements for modern cosmetic ingredients include their multifunctionality, safety and effectiveness. The confirmed biological effect of an active molecule is usually not sufficient to obtain the innovative cosmetic formulation. Numerous physicochemical properties of a biological active compound are decisive to ensure an overall utility following topical application [18]. Bioavailability and skin permeability are restricting factors for the cosmetic use of many potential active substances [19]. The application of natural compounds as cosmetic ingredients takes on a new meaning in the case of natural extracts, which contain a mixture of molecules with different physicochemical characters. According to this fact, there is still a question about the comparison of the skin absorption of pure compounds like *E*-resveratrol or *E*-ε-viniferin with the mixtures rich in stilbenoids (e.g., GCE). The physicochemical character of a pure substance is the crucial parameter which can restrict its permeability. From another point of view, the application of such mixtures would ensure a wide range of biological activities from the surface of the stratum corneum, through the epidermis to deep layers of the dermis.

In this study, we evaluated the cosmetic potential of GCE from previously selected polyphenol-rich cultivars [20] as multifunctional rejuvenating agents through two different mechanisms; (1) skin whitening via tyrosinase inhibition using enzymatic assays and docking data, (2) delaying of cellular senescence using sirtuin activation assays. Activities of pure constituents of GCE like *E*-resveratrol and *E*-ε-viniferin were compared to GCE, a natural biosourced blend of stilbenoids. Finally, we evaluated the availability of the major constituents of GCE and their capacity to penetrate the skin barrier.

## 2. Results and Discussion

### 2.1. Concentration of Polyphenols in GCE

**Figure 1:**
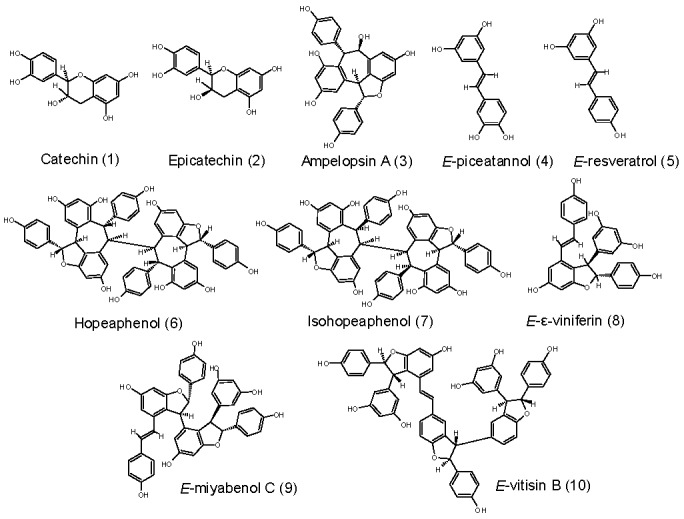
The structures of major polyphenols analyzed in GCE.

The concentration of major polyphenols present in GCE from five previously selected cultivars were quantified by HPLC analyses (Table 1) [20]. Ten major compounds were identified: two flavonoids (catechin, epicatechin) as well as eight stilbenoids (ampelopsin A, *E*-resveratrol, *E*-piceatannol, hopeaphenol, isohopeaphenol, *E*-ε-viniferin, *E*-miyabenol C and *E*-vitisin B). The chemical structures of the compounds are presented in Figure 1.

Eight polyphenols were identified by their comparison with pure standards i.e., *E*-resveratrol, *E*-piceatannol, catechin, epicatechin, *E*-ε-viniferin, hopeaphenol, ampelopsin A and *E*-vitisin B. Two compounds (isohopeaphenol and *E*-miyabenol C) were assigned according to elution order, UV spectra and MS data from the literature [21]. HPLC analyses showed very high concentrations of total polyphenols in GCE from the five selected cultivars ranging from 16.8% ± 7.4% for Sauvignon to 39.4% ± 3% for Villard Noir. Savagnin blanc GCE contained the highest concentration in *E*-resveratrol (12.0% ± 4.4%), while Villard Noir GCE was characterized by the highest contents in *E*-ε-viniferin (3.6% ± 0.2%) and *E*-vitisin B (17.2% ± 1%). These varietal-specific polyphenol compositions might drive various levels of cosmetic activities.

### 2.2. Sirtuin Activation

**Figure 2:**
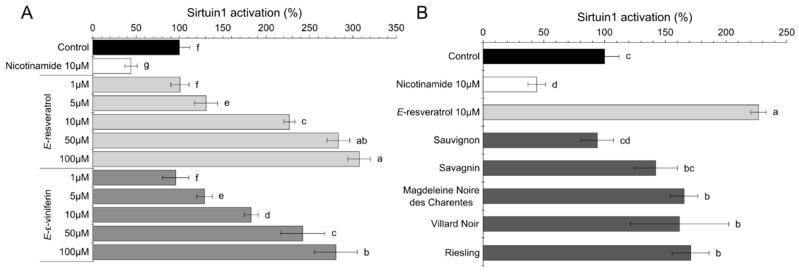
Sirtuin activation results for pure stilbenoids (*E*-resveratrol, *E*-ε-viniferin) and nicotinamide (**A**) and for the GCE (50 µM) from five cultivars (**B**). Data are mean values ± standard deviation (*n* = 3). Different letters indicate significant difference between means at *p* < 0.001.

As a first step, we evaluated the anti-aging action of GCE from the five cultivars (Villard Noir, Sauvignon, Savagnin, Riesling and Magdeleine Noire des Charentes). Figure 2A presents the results of sirtuin activation by two pure stilbenoids (*E*-resveratrol and *E*-ε-viniferin) in a 1–100 µM concentration range in comparison with nicotinamide as the negative control.

As shown in Figure 2A, compared to the control sample, the efficient concentration of both sirtuin activators (*E*-resveratrol and *E*-ε-viniferin) was at least 5 µM and reached a 3-fold increase at 100 µM. *E*-resveratrol was prompt to slight higher SIRT1 activation (from 130% ± 13% for 5 µM to 307% ± 30% for 100µM) in comparison to *E*-ε-viniferin (95% ± 15% and 280% ± 24% respectively).

It is already known that *E*-resveratrol exhibits a beneficial activity for the human organism through the activation of SIRT1. Howitz et al., 2003 [16] reported that *E*-resveratrol lowers the Michaelis Menten constant of SIRT1 and increases cell survival by stimulating SIRT1-dependent deacetylation of p53. In yeast, *E*-resveratrol mimics calorie restriction by the stimulation of SIR2, increasing DNA stability and extending lifespan by 70%. Stacchiotti et al. (2016) [22] confirmed that the first function of *E*-resveratrol is to reduce inflammation and to limit oxidative damage in tissues. The antiaging properties of *E*-resveratrol via SIRT1 activation are also associated to oxidative metabolism improvement in crucial organs like the heart, vessels, muscles and kidney [22]. In other studies, in the presence of a SIRT1 inhibitor like nicotinamide, *E*-resveratrol stimulated SIRT1 [16]. Moreover, concentration-dependent effects were observed for its activity. What is more, by the stimulation of SIRT1-dependent deacetylation of p53, the *E*-resveratrol molecule increases cell survival in adverse conditions [16]. It has also been demonstrated that *E*-resveratrol has the ability to protect human cells from lipid damage, which can be significant for the prevention of lipophilic skin barrier structures degradation. Although the complete mechanism of *E*-resveratrol activity still remains to be fully explained by further studies, the sirtuin activation is the main activity of *E*-resveratrol in establishing its various health benefits [23]. Despite a well described sirtuin activation by *E*-resveratrol [1,16], very little is still known about the effect of *E*-ε-viniferin. The protective role of *E*-ε-viniferin was described by Fu et al. (2012) [24] in Huntington Disease cell models. It was demonstrated that *E*-ε-viniferin decreases the level of Reactive Oxygen Species (ROS) and prevents loss of mitochondrial membrane potential in cells expressing the mutant Huntington protein. The expression of this protein results in the decreased deacetylase activity of SIRT3, and as a result, leads to reduction in cellular NAD(+) levels and mitochondrial biogenesis in cells. According to the studies, *E*-ε-viniferin activates AMP-activated kinase and enhances mitochondrial biogenesis [24]. In our research, we demonstrated that *E*-ε-viniferin was a sirtuin activator equivalent to *E*-resveratrol. We further tested the potential of GCE polyphenol-rich natural extract to activate SIRT1. Most of the GCE (Riesling, Magdeleine Noire, Villard Noir and Savagnin) showed a relatively high SIRT1 activation compared to the control sample. Riesling was the most promising cultivar, with 171% SIRT1 activation, followed by Magdeleine Noire (165%), Villard Noir (162%) and Savagnin (142%). Only GCE from Sauvignon showed no induction effect for SIRT1. SIRT1 activation by GCE from Magdeleine Noire, Villard Noir and Savagnin were at least equivalent to activation by 5 µM *E*-resveratrol. First sirtuin activators were discovered for SIRT1 in 2003, and the most potent was *E*-resveratrol [1]. Several studies also described SIRT1 activation by botanical extracts. Corbi et al., (2018) reported promising results for lemon beebrush (*Lippia citriodora*), radish (*Raphanus sativus*) and tomato (*Solanum lycopersicum*) extracts [25]. Wang et al. also reported the sirtuin induction activity of Traditional Chinese medicines. Milkvetch (*Astragalus membranaceus*), Chinese ginseng (*Panax ginseng*) and three-seven root (*Panax notoginseng*) have been reported to exert protective effects against oxidative stress in mitochondria. The results showed that those extracts enhanced the deacetylated activity of SIRT1 and inhibited intracellular reactive oxygen species formation [26]. Then, GCE represent promising natural ingredients for skin rejuvenation, especially in comparison with the strong SIRT1 activators like *E*-resveratrol and *E*-ε-viniferin. It has been shown that sirtuin regulation by *E*-resveratrol and its derivatives act via complex direct interactions in an isoform-specific manner [27]. Resveratrol inhibits human SIRT3 and stimulates SIRT5 and SIRT1 depending on complex bindings with the catalytic pocket.

### 2.3. Tyrosinase Inhibition

#### 2.3.1. Enzyme Assay

**Figure 3:**
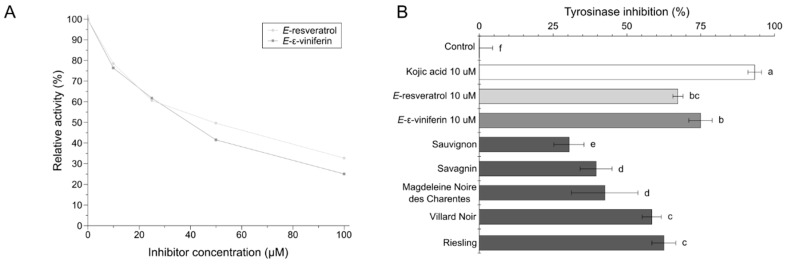
Inhibition of tyrosinase by *E*-ε-viniferin and E-resveratrol (**A**) and by GCE (50 µM) from the selected cultivars (**B**). Data are mean values ± standard deviation (*n* = 3). Different letters indicate significant difference between means at *p* < 0.001.

Figure 3A presents tyrosinase inhibition by two pure stilbenoids (*E*-ε-viniferin and *E*-resveratrol) and Figure 3B shows tyrosinase inhibition by GCE from selected cultivars in comparison to kojic acid, *E*-resveratrol and *E*-ε-viniferin as positive reference compounds.

As shown in the Figure 3B, all the tested GCE as well as *E*-resveratrol, *E*-ε-viniferin are relatively active tyrosinase inhibitors. The highest potential was shown for *E*-ε-viniferin (76% ± 2%) and *E*-resveratrol (75% ± 4%). GCE presented various capacities to inhibit tyrosinase. Riesling and Villard Noir GCE were the most active with inhibition levels of 62.5% and 58.5%, respectively. Magdeleine Noire des Charente GCE (42.5%) and Savagnin GCE (39.5%) also exhibited relatively strong tyrosinase inhibition activity, whereas Sauvignon GCE was less effective, however with a quite efficient inhibition level (30.4%). *Vitis vinifera* L. leaf extracts were already mentioned in the literature as natural sources of tyrosinase inhibitors [28]. An *E*-resveratrol derivative, oxyresveratrol, was shown to inhibit browning in cloudy apple juices at a concentration as low as 0.01%. It was found that this stilbenoid was about 0.2% more potent than kojic acid [29].

Although grape canes accumulated much more stilbenoids than grape leaves, tyrosinase inhibition assays on grape canes are unprecedented. Tyrosinase inhibition of GCE in comparison to reference compounds with known whitening activity like *E*-resveratrol and *E*-ε-viniferin confirmed the potential of GCE as novel cosmetic active ingredients. The present study showed that all GCE tested were very potent tyrosinase inhibitors which is crucial for considering their potential as skin whitening agents.

#### 2.3.2. IC_50_ Determination

**Figure 4:**
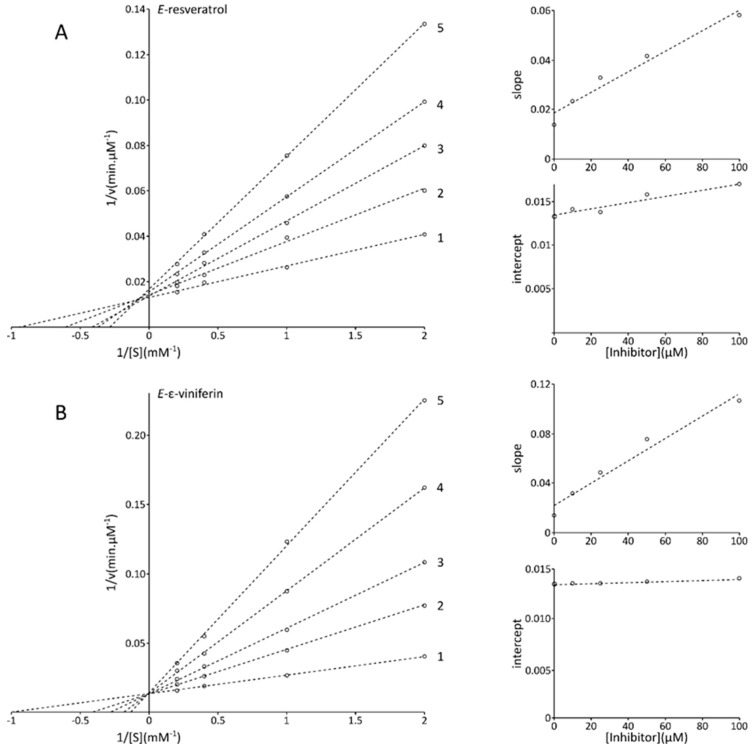
Lineweaver–Burk plots for inhibition of mushroom tyrosinase by *E*-resveratrol (**A**) and *E*-ε-viniferin (**B**). Concentrations of *E*-resveratrol (**A**) and *E*-ε-viniferin for curves 1–5 were 0, 10, 25, 50 and 100 µM, respectively. The inset represents the secondary plots representing slopes of the double reciprocal plot versus the concentrations of each inhibitor (i.e., *E*-resveratrol (**A**) and *E*-ε-viniferin (**B**)) used for the determination of *K*_I_ and secondary plot of intercepts of the double reciprocal plot versus the concentrations of each inhibitor (i.e., *E*-resveratrol (**A**) and *E*-ε-viniferin (**B**)) used for the determination of *K*_IS._

The kinetic behavior of mushroom tyrosinase during inhibition by *E*-resveratrol and *E*-ε-viniferin was studied. The kinetic parameters for mushroom tyrosinase obtained from a Lineweaver–Burk plot for inhibition by *E*-resveratrol (Figure 4A, line 1) show that *K*m was equal to 1.02 (0 µM, control) to 3.43 (100 µM) mM and *V*max was equal to 74.63 µM/min (0 µM, control) versus a mean equal to 66.38 ± 6.44 µM/min for the different tested *E*-resveratrol concentrations. A Lineweaver–Burk plot for inhibition by *E*-ε-viniferin (Figure 4B, line 2) shows that *K*m was equal to 1.02 (0 µM, control) to 7.63 (100 µM) mM and *V*max was equal to 74.63 µM/min (0 µM, control) versus a mean equal to 73.01 ± 1.14 µM/min for the different tested *E*-resveratrol concentrations. The results presented in Figure 4 showed that both *E*-resveratrol and *E*-ε-viniferin are competitive inhibitors because increasing the concentration of the compounds resulted in a line with common intercept on the 1/v axis but with different slopes. The inhibition constants of each inhibitor, *K*_I_ for binding to the free enzyme (to form EI complex), and *K*_IS_ for the binding to the enzyme–substrate complex (to form ESI complex) were determined using the secondary plot and the secondary replot (Figure 4), respectively. The secondary plots representing slopes (*K*m/*V*max) of the double reciprocal plots against the inhibitor concentrations allowed us to calculate an EI dissociation constant (*K*_I_) of 46.25 and 24.22 µM for *E*-Resveratrol and *E*-ε-viniferin, respectively. The secondary replots, representing intercepts of the double reciprocal relation against the inhibitor concentrations, allowed us to calculate an ESI dissociation constant (*K*_IS_) of 364.86 and 2355.73 µM for *E*-resveratrol and *E*-ε-viniferin, respectively. Therefore, if these calculations proposed a putative binding that could occur either to the free tyrosinase enzyme or to tyrosinase enzyme associated with its substrate (the present results with 7.9- and 97.3-times higher values of *K*_IS_ for *E*-resveratrol and *E*-ε-viniferin, respectively), these results strongly suggested a much weaker binding affinity to the tyrosinase enzyme–substrate complex rather than to the free tyrosinase enzyme, thus indicating that the predominant inhibition mechanism of each inhibitor is competitive.

We calculated IC_50_ values of 52.93 µM for *E*-ε-viniferin and 60.75 µM for *E*-resveratrol. These IC_50_ values were within ranges to those reported in the literature [13].

The present study established that *E*-resveratrol and *E*-ε-viniferin inhibit the enzyme very effectively. GCE also exhibited tyrosinase inhibition at a relatively high level. Previous reports showed that *E*-ε-viniferin is the most active tyrosinase inhibitor, with an IC_50_ = 4.1 μM. It is four times more potent than kojic acid (IC_50_ = 16.9 μM), and 62-fold more active than ascorbic acid (IC_50_ = 255 μM) to inhibit tyrosinase. *E*-resveratrol has a moderate inhibitory activity (IC_50_ = 52.8 μM), quite similar to arbutin (IC_50_ = 55.1 μM) [12].

### 2.4. Molecular Docking for the Binding of E-resveratrol and E-ε-viniferin with Tyrosinase

**Figure 5:**
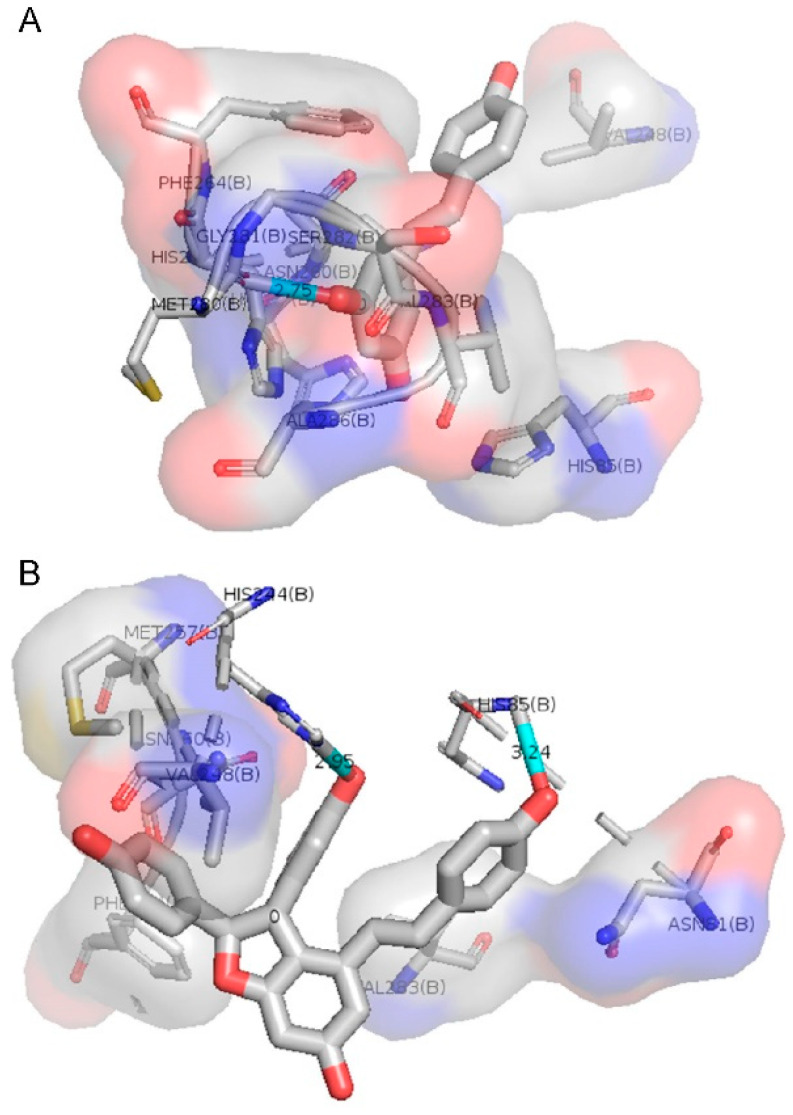
Molecular docking simulations of binding tyrosinase and *E*-resveratrol (**A**) and *E*-ε-viniferin (**B**).

Figure 5 presents the docking data made for *E*-resveratrol and *E*-ε-viniferin. The results of the docking data clearly indicate that *E*-resveratrol and *E*-ε-viniferin exhibited tyrosinase inhibition potential. However, the affinity for *E*-ε-viniferin is slightly higher with a calculated affinity of −7.73 versus −5.95 kcal/mol as a consequence of interactions with His85 and His244 through hydrogen bounds and π-π stacking versus only one hydrogen bound interaction with Met280 for *E*-ε-viniferin versus *E*-resveratrol, respectively. Both affinities were stronger for these two stilbenoids than the one observed for kojic acid (−5.7 kcal/mol [30]), as well as for glabridin (−7.15 kcal/mol [31]) using a similar docking approach. Compared with l-DOPA, these two stilbenoids bind to tyrosinase at the same site, thus confirming their competitive inhibition mechanism [31]. Affinities for these two stilbenoids were in the range of that observed for l-DOPA (i.e., −6.98 kcal/mol [31]).

The results confirmed the previous enzyme assay outcomes, where *E*-ε-viniferin was a more potent tyrosinase inhibitor than *E*-resveratrol.

Tyrosinase dysfunctions advance with aging and can lead to malignant melanoma, as well as pigmentary disorders such as freckles or melisma [17]. *E*-resveratrol and *E*-ε-viniferin-rich GCE are a good alternative as a natural source of these stilbenoids for the prevention of some pigmentation diseases.

### 2.5. Bioavailability and Skin Permeability Potential

**Figure 6:**
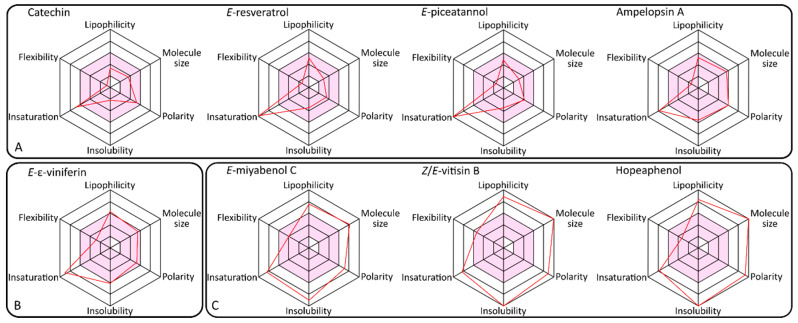
Availability of major GCE constituents to penetrate stratum corneum. (**A**) most potent permeability, (**B**) medium permeability, (**C**) less potent permeability.

Figure 6 presents the crucial physicochemical properties of major constituents of GCE that determine their overall bioavailability and skin permeability.

The permeability evaluation evaluates at first glance the ability of skin penetration of a molecule. Pink central zones in the radar charts of Figure 6 represent the optimal range for each property. The partition coefficient logarithm log*P* (lipophilicity) should obtain values between −0.7 and +5.0, molecular weight (molecule size) should be between 150 and 500 g/M, polarity (topological polar surface area, TPSA) should range between 20 and 130 Å^2^, solubility (log S) should be not higher than 6, saturation (fraction of carbons in the sp3 hybridization) should be not less than 0.25 and flexibility no more than 9 rotatable bonds [32]. Based on these calculations, it was evaluated that GCE molecules most likely to be absorbed in body are catechin and epicatechin (with the same physicochemical characteristics), *E*-resveratrol, *E*-piceatannol as well as ampelopsin A. These molecules were characterized by proper molecular size, electron distribution, polarity and molecule character. Molecules characterized by higher molecular weight like *E*-miyabenol C, *E*-vitisin B, as well as hopeaphenol and isohopeaphenol (with the same physicochemical characteristics), from a physicochemical point of view, are not able to be assimilated as they hardly overcome the physical barriers like membranes, as well as dermis. *E*-ε-viniferin fulfills the majority of the rules required for good skin permeability. The polarity, lipophilicity, insolubility, flexibility and molecular size of the molecules were estimated by mathematical equations. Worth pointing out is the fact that the calculations are only estimating methods for the overall skin permeability, and dermal penetration tests will be required to confirm predictions.

During the past decades, different rule sets were established, helping to define suitable predictions of drug absorption. The most popular criteria are Lipinski’s rule of five [33], but several other approaches are also available [34,35,36,37]. Table 2 presents the information about the compliance of GCE polyphenols with common known bioavailability rules: Lipinski (MW < 500, log*P* < 4.15, number of N or O atoms < 10, number of N or OH groups < 5) [33], Ghose (160 < MW < 480, −0.4 < log*P* < 5.6, 40 < MW < 130, 20 < number of atoms < 70) [34], Veber (number of rotatable bonds < 10, TPSA < 140) [35], Egan (log*P* < 5.88, TPSA < 131.6) [36] and Muegge (200 < MW < 600, −2 < log*P* < 5, TPSA < 150, numbers of rings < 7, number of carbons > 4, number of heteroatoms > 1, number of rotatable bonds < 15, NHA < 10, NHD < 5) [37].

Based on these calculations, it can be indicated with relatively high probability which structures are most likely to penetrate the skin barrier. GCE polyphenols characterized with MW above 500 g/mol (hopeaphenol, isohopeaphenol, *E*-miyabenol C and *E*-vitisin B) are simultaneously too lipophilic (log*P* > 5) and characterized by improper electron distribution (TPSA > 140) as well as hydrogen bonding characteristics (NHD > 5 or NHA > 10). Moreover, molecular volumes of these molecules can limit their skin absorption and, from a spatial point of view, their molecular bond violations would additionally restrict their ability to be active within the skin structures. Such compounds, after topical application, remain as a residue on the surface or, depending on skin conditions, penetrate only to outer layers of hydrophobic stratum corneum. Skin barrier properties are based on lipid bilayers. The successful transdermal drugs have been limited by parameter thresholds even more restrictive than the Rule of Five [19]. Therefore, newly available statistical calculations explain very accurate rules for transdermal routes of active substances. They base mainly on their physicochemical parameters. The new thresholds for current transdermal drugs are MW  <  335, NHD  ≤  2, NHA  ≤  5, and log*P* < 5 [19].

Despite their inability to overcome the stratum corneum barrier, high molecular stilbenoids (trimers and tetramers of resveratrol) still remain of high interest due to their beneficial activities for the skin. As already shown in many studies, these metabolites exhibit strong antioxidant properties [38], and in parallel, due to their character, they are distinguished by high compartment to intercellular cement components and the lipophilic protective barrier of the skin. In cases of harmful conditions and over-dried skin, these polyphenols can play an important role as active emollients with regenerative and antioxidant ability on the skin’s surface [39].

Considering the above-mentioned rules for skin penetration, the most potent candidates for cosmetic active ingredients are then catechin, epicatechin, *E*-piceatannol, *E*-resveratrol and *E*-ε-viniferin. Our calculations predict that these polyphenols can overcome skin barrier and act within the structure of the dermis, which is especially important regarding their activity for enzymatic functions of the skin. Confirmed ability to modulate tyrosinase and sirtuin activity and their good prediction for skin penetration allow the low-molecular stilbenoids (monomers and dimers of resveratrol) like *E*-resveratrol and *E*-ε-viniferin to act as rejuvenating and whitening agents. In addition, their high antioxidant activity ensures multi-level effects for skin cells. GCE are blends of polyphenols, which exhibit various biological activities and are characterized by different physicochemical character properties. Therefore, the extracts ensure multidirectional therapeutic effects as well as effective protection for human skin.

Therefore, it can be claimed that polyphenol-rich GCE would be more beneficial in skincare treatments than encapsulated pure stilbenoids [22]. Further experiments on skin models are still required to confirm the potential of GCE as a natural multifunctional ingredient for eco-friendly dermocosmetics.

## 3. Materials and Methods

### 3.1. Chemicals and Reagents

*E*-resveratrol and other standards were purchased from Sigma-Aldrich (St. Louis, MO, USA). *E*-ε-viniferin was purified from grape canes as previously described [40]. Mushroom tyrosinase solution and L-DOPA were obtained from Sigma-Aldrich (St. Louis, MO, USA). Ultrapure water was obtained from a Millipore Milli-Q water purification system (Merck Millipore, city, Germany).

### 3.2. Plant Material

Grape canes from five selected varieties (Villard Noir, Sauvignon, Savagnin, Riesling and Magdeleine Noire des Charentes) were harvested in January 2016 at the INRA grape repository at “Domaine de Vassal” (34340 Marseillan-Plage, France: http://www.1.montpellier.inra.fr/vassal). Twenty-five grape stalks were harvested for each variety after the pruning of five stalks from five different vines. Grape stems were cut into 10 cm long sections and stored for 10 weeks at 20 °C in the dark, enabling *E*-resveratrol and *E*-piceatannol post-harvest accumulation. Then, grape stems were ground at first with a cooled analytical grinder (Ika-Werke, Staufen, Germany) and additionally with a cutting mill (Polymic PX-MFC 90 D, Kinematica AG, Lucerne, Switzerland) to obtain 1 mm-sized particles. The powder was lyophilized and stored at −20 °C until extraction [41]. In total, 20 g of dried powder was extracted with 500 mL of ethanol/water mixture (60/40; *v*/*v*). Samples were extracted for 45 min in reflux at 83 °C and filtered. Then, supernatants were evaporated using a Heidolph 94200 rotavapor (Bioblock, Schwabach, Germany) coupled with a vacuum pump (Vacuubrand PC500 series, Wertheim, Germany). The resulting extracts were lyophilized, giving dried GCE available for further in vitro assays.

### 3.3. HPLC Analyses

The HPLC system was made of a Waters 717 plus Autosampler, a Waters 996 photodiode array detector and a Waters 600 Controller (Waters, Milford, MA, USA) pump and was controlled by Empower 2 software (Waters, Milford, MA, USA). We achieved analyte separation through the injection of 20 µL of extracts on a column packed with 3 µm particles (250 × 4 mm, Multospher 120 RP18HP; CS-Service, Langerwehe, Germany) at 24 °C. The mobile phase was made of 0.1% phosphoric acid (solvent A) and acetonitrile (solvent B) pumped at 0.5 mL min^−1^. We employed a linear gradient that started at 5% B and increased to 72.5% in 60 min. Quantification was done using pure standards using a five-point calibration curve (0–100 ppm) in Maxplot detection mode. The isohopeaphenol was quantified using hopeaphenol calibration curve.

### 3.4. Sirtuin Activation

Sirtuin-activation (SIRT1) evaluation was performed using GCE at 50 µg/mL in comparison with 10 µM of pure *E*-resveratrol and *E*-ε-viniferin (as activators) and nicotinamide (as the inhibitor). SIRT1 activity was determined using the SIRT1 Assay Kit (Sigma-Aldrich, St. Louis, MO, USA) following manufacturer instructions and using a fluorescent spectrometer (Biorad VersaFluor, Marnes-la-Coquette, France) set with 340 nm excitation and 430 nm emission wavelengths. The relative SIRT1 activity was revealed as a relative percentage to the corresponding control (adding the same volume of extraction solvent) for each extract.

### 3.5. Tyrosinase Inhibition

#### 3.5.1. Enzyme Assay

The tyrosinase inhibition assay was measured as described by Neely et al. (2009) [42]. Each 1 mL assay contained a final concentration of 100 mM sodium phosphate (pH 6.5) and 2 mM L-DOPA. Finally, 0.2 mg/mL of mushroom tyrosinase solution (Sigma-Aldrich) was added to the mixture. Control, with an equal amount of extraction solvent replacing the extract, was routinely carried out. Reaction processes were traced by using a microplate reader (BioTek ELX800; BioTek Instruments Inc., Winooski, VT, USA) at a wavelength of 475 nm. The tyrosinase inhibitory effect was expressed as a % of inhibition relative to the corresponding control for each extract. Standard compound concentrations applied in a study were 100 µM and the concentration of tested extracts was 50 µg/mL. The experiments were repeated three times and the average results with standard deviation values were given in Figure 3B.

#### 3.5.2. IC_50_ Determination

To assume the range of inhibitor amounts needed for calculation of the IC_50_ value, various concentrations of *E*-resveratrol and *E*-ε-viniferin were used. The assays used for the calculations were prepared within the range of inhibitors from 1 to 100 µM. In case of a significant change in the shape of the activity–time curve, the rates were calculated based on steady state rate regions. The concentration of inhibitor causing the 50% tyrosinase activity inhibition was extrapolated from % activity–inhibitor curves [42]. The experiments were repeated three times and the average of the IC_50_ values were given in Figure 4A and 4B.

### 3.6. Docking Data for the Binding of E-resveratrol and E-ε-viniferin with Tyrosinase

Molecular docking simulation of *E*-resveratrol and *E*-ε-viniferin was performed with Ligplot+ software (European Bioinformatics Institute, Cambridge, UK), autodock Vina (The Scripps Research Institute, La Jolla, CA, USA) and Pymol v2.1.1 (Schrodinger, New York, NY, USA) to predict the conformation of these molecule ligands within the appropriate target binding site of tyrosinase (PDB: 2Y9X).

### 3.7. Bioavailability and Skin Permeability Potential

Drug-likeness potential as well as skin permeability, as crucial factors for active molecule effectiveness, may be defined as a complex balance of various physicochemical properties and structure features which determine whether the molecule is similar to the known drugs. These properties, mainly hydrophobicity, electronic distribution, hydrogen bonding characteristics, molecule size, flexibility and the presence of various pharmacophoric features, influence the behavior of molecules in a living organism, including bioavailability, transport properties, affinity to proteins, reactivity, toxicity, metabolic stability and many others [43]. Simple count criteria (like limits for molecular weight, log*P* or number of hydrogen bond donors or acceptors) have also relatively limited applicability and are useful only to discard some of the potential active molecules from further research [19]. The Molecular Polar Surface Area (TPSA) values for all of the structures were calculated based on the methodology published by Ertl et al. [44] as a sum of fragment contributions in the whole molecule. O- and N-centered polar fragments were also considered. Method for the calculation of molecule volume as well as partition coefficient logarithm (log*P*) values were developed using SwissADME [32]. 3D molecular geometries for a training set were fully optimized by the semi-empirical AM1 method [45,46]. Lipinski’s “Rule of Five” states that most “drug-like” molecules have log*P* ≤ 5, molecular weight ≤ 500, number of hydrogen bond acceptors ≤ 10, and number of hydrogen bond donors ≤ 5. Molecules violating more than one of these rules may have very low or no bioavailability at all [33]. Biological activity prediction calculations are based on Bayesian statistics to compare structures of representative ligands active on the particular target with structures of inactive molecules and to identify substructure features (which in turn, determine physicochemical properties) typical for active molecules [47].

## 4. Conclusions

Polyphenol-enriched GCE were able to activate SIRT1 at similar levels than 5 µM *E*-resveratrol or *E*-ε-viniferin. Skin whitening potential via tyrosinase inhibition assay showed that GCE capacities were comparable to pure *E*-resveratrol and *E*-ε-viniferin. Especially, Villard Noir and Riesling GCE could be useful as skin-lightening agents and may be used against dark spots in dermocosmetics. Additionally, drug-likeness of GCE components showed various capability in skin permeation, providing sufficient effectiveness in different dermis structures. Skin physiological processes supported by these active compounds ensure the right skin barrier functions as well as efficient skin tissues recovery. In conclusion, the potential application of GCE is of enormous interest not only for industry but also for consumers that increasingly demand for natural ingredients, which are required in the so called “eco cosmetics”.

## Figures and Tables

**Table 1:** Concentration of major polyphenols contained in grape cane extracts (GCE) from selected cultivars (% of dry GCE). (1) catechin, (2) epicatechin, (3) ampelopsin A, (4) *E*-piceatannol, (5) *E*-resveratrol, (6) hopeaphenol, (7) isohopeaphenol, (8) *E*-ε-viniferin, (9) *E*-miyabenol C, (10) *E*-vitisin B.

Cultivars	Compounds (% of GCE)										
	1	2	3	4	5	6	7	8	9	10	Total
Magdeleine Noire des Charentes	1.3 ± 0.3	1.0 ± 0.6	1.8 ± 0.5	8.1 ± 3.6	4.4 ± 0.6	1.2 ± 0.2	0.5 ± 0.1	2.8 ± 0.2	0.4 ± 0.1	5.2 ± 2.6	26.6 ± 8.8
Riesling	0.6 ± 0.1	1.0 ± 0.2	1.9 ± 0.2	3.3 ± 0.4	4.7 ± 1.1	2.9 ± 0.6	0.3 ± 0.1	1.7 ± 0.1	3 ± 0.2	7.6 ± 4.0	27.1 ± 7.2
Savagnin Blanc	1.9 ± 0.2	2.1 ± 0.1	2.2 ± 0.3	12.0 ± 4.4	7.2 ± 2.0	1.3 ± 0.2	0.5 ± 0.2	3.2 ± 0.7	0.3 ± 0.1	5.1 ± 2.4	35.7 ± 10.6
Sauvignon	1.2 ± 0.1	0.8 ± 0.5	0.7 ± 0.3	5.0 ± 1.9	1.3 ± 0.5	1.3 ± 0.1	0.1 ± 0.1	2.5 ± 0.8	0.3 ± 0.1	3.8 ± 2.9	16.8 ± 7.4
Villard Noir	0.6 ± 0.0	1.4 ± 0.2	0.3 ± 0.1	6.7 ± 1.0	6.7 ± 0.1	1.4 ± 0.2	1.3 ± 0.1	3.6 ± 0.2	0.2 ± 0.1	17.2 ± 1.0	39.4 ± 3.0

**Table 2:** Bioavailability levels for GCE components according to their physicochemical properties (A) most potent, (B) medium, (C) less potent to penetrate stratum corneum.

Bioavailability Level	Polyphenol	Compliance with the Bioavailability Rules				
		Lipinski [33]	Ghose [34]	Veber [35]	Egan [36]	Muegge [37]
A	catechin	yes	yes	yes	yes	yes
A	epicatechin	yes	yes	yes	yes	yes
A	*E*-resveratrol	yes	yes	yes	yes	yes
A	*E*-piceatannol	yes	yes	yes	yes	yes
A	ampelopsin A	yes	yes	yes	yes	no
B	*E*-ε-viniferin	yes	no	yes	yes	no
C	hopeaphenol	no	no	no	no	no
C	isohopeaphenol	no	no	no	no	no
C	*E*-miyabenol C	no	no	no	no	no
C	*E*-vitisin B	no	no	no	no	no

## Abbreviations

GCE	grape cane extracts
log*P*	partition coefficient logarithm
MW	molecular weight
NHA	number of hydrogen acceptors
NHD	number of hydrogen donors
TPSA	topological polar surface area
